# Total Sleep Time Severely Drops during Adolescence

**DOI:** 10.1371/journal.pone.0045204

**Published:** 2012-10-17

**Authors:** Damien Leger, François Beck, Jean-Baptiste Richard, Emmanuelle Godeau

**Affiliations:** 1 Université Paris Descartes, Sorbonne Paris Cité, APHP (Assistance Publique Hôpitaux de Paris), Hôtel Dieu, Centre du Sommeil et de la Vigilance, Paris, France; 2 INPES (Institut national de Prévention et d'éducation pour la santé), Direction des Affaires Scientifiques, Cermes 3, Equipe Cesames (centre de recherche médecine, sciences, santé, santé mentale, société, Université'Paris Descartes, CNRS UMR 821, Inserm U988/EHESS), Saint Denis, France; 3 UMR 1027 INSERM/Toulouse III, University Paul Sabatier, Toulouse and Service Médical du Rectorat, Toulouse, France; University of Pennsylvania School of Medicine, United States of America

## Abstract

Restricted sleep duration among young adults and adolescents has been shown to increase the risk of morbidities such as obesity, diabetes or accidents. However there are few epidemiological studies on normal total sleep time (TST) in representative groups of teen-agers which allow to get normative data.

**Purpose:**

To explore perceived total sleep time on schooldays (TSTS) and non schooldays (TSTN) and the prevalence of sleep initiating insomnia among a nationally representative sample of teenagers.

**Methods:**

Data from 9,251 children aged 11 to 15 years-old, 50.7% of which were boys, as part of the cross-national study 2011 HBSC were analyzed. Self-completion questionnaires were administered in classrooms. An estimate of TSTS and TSTN (week-ends and vacations) was calculated based on specifically designed sleep habits report. Sleep deprivation was estimated by a TSTN – TSTS difference >2 hours. Sleep initiating nsomnia was assessed according to International classification of sleep disorders (ICSD 2). Children who reported sleeping 7 hours or less per night were considered as short sleepers.

**Results:**

A serious drop of TST was observed between 11 yo and 15 yo, both during the schooldays (9 hours 26 minutes vs. 7 h 55 min.; p<0.001) and at a lesser extent during week-ends (10 h 17 min. vs. 9 h 44 min.; p<0.001). Sleep deprivation concerned 16.0% of chidren aged of 11 yo vs. 40.5% of those of 15 yo (p<0.001). Too short sleep was reported by 2.6% of the 11 yo vs. 24.6% of the 15 yo (p<0.001).

**Conclusion:**

Despite the obvious need for sleep in adolescence, TST drastically decreases with age among children from 11 to 15 yo which creates significant sleep debt increasing with age.

## Introduction

Sleep is recognized as a major contributing factor to physical and mental health in humans, especially in young adults. Although the precise functions of sleep are not entirely understood, crucial studies have demonstrated its critical role in many important somatic, cognitive, and psychological processes. For example, it is believed that sleep is beneficial for energy conservation, neuronal recuperation, and brain plasticity [1]–[4]. Sleep has been shown to play a major role in the metabolic, cardiovascular, respiratory, immune and thermoregulatory processes which contribute to daytime brain functioning and body homeostasis. Sleep is also essential for growth, as growth hormone is selectively secreted during slow wave sleep [1]–[2], [4]–[5]. Beside these metabolic functions, sleep is mainly involved in cognitive and psychological processes, including learning and memory consolidation, as well as emotional memory and processing [6]–[11].

Acute and chronic sleep deprivation studies have reinforced the belief that sleep is essential to good physical and mental health. Chronic sleep deprivation, (defined as sleeping 50%–75% of a normal 8 h night's sleep for several successive nights) alters vigilance, cognition and mood, causes anxiety and increases the risk of accidents [12]–[14]. Chronic ‘too short sleep’ (less than 6 hours per 24 hours in adults) is associated with an elevated risk of diabetes, overweight, and cardiovascular diseases [15]–[17]. Acute sleep deprivation (defined as sleeping 25–50% of a normal 8 h night's sleep) contributes to increased inflammation and disturbs the immunological response [18]–[20]. As adolescence is considered a particularly vulnerable period during which physiological, psychological and cognitive processes undergo maturation, it could be hypothesised that short sleep and sleep deprivation would have a worse effect in this group than in young adults both in the short and in the long terms.

However, there are several studies, mostly made in urban settings, showing that teens have frequently a too short amount of sleep during week-days: In Taiwan, 1939 adolescent aged from 12 to 16 years old have been interviewed at schools in the Lin-Kou district [21]. Their mean (SD) sleep duration on weekdays was 7.35 (1.23) hours and 9.38 (1.62) hours on weekends. Weekday's night sleep decreased significantly when school grade increased (6.87 hours (1.14) for high school seniors). In Iceland, a group of 668 adolescents (aged 11, 13, and 15) were followed longitudinally three times, every 5 years. Iceland teens had delayed bedtimes and shorter nocturnal sleep during the week increasing with age [22]. The HELENA study also interviewed adolescents from 10 European cities in Austria, Belgium, France, Germany, Greece, Hungary, Italy, Spain and Sweden about their sleep duration. It found a rather low averaged total sleep time around 8 hours per day [23]. In Korea, a 2638 sample of urban high school students, aged from 14 to 17 years old (yo), self-reported sleep schedules and habits during weekdays and week-ends and performed attention tasks [24]. Their mean (SD) sleep duration on weekdays was 5 hours 42 minutes (1 hour 0 minutes) per day and on weekends: 8 hours 24 minutes (1 hour 36 minutes) per day. In Brazil, short sleep duration was also found increased with age in a 4452 adolescents group of Pelotas (aged 10–12 years) participating in a prospective birth cohort [25]. Using less than 6 hours to define short sleep, a longitudinal one year survey of 4,175 adolescents aged from 11 to 17years old living in Texas, found an impressive rate of 20% of short sleepers [26]. Conversely, a Canadian longitudinal sleep study testing the link between body mass index (BMI) and total time spent in bed (according to mother's testimony), found a rather higher time spent in bed, in the 10 yo children [27]. They were estimated to stay in bed a mean (SD) time of 10.2 (0.6) hours at 10 yo and 9.9 (0.6) hours at 13 yo.

All these studies showed that short sleep was common in adolescents in many parts of the world. However defining short sleep or sleep deprivation in adolescents is made difficult by the limited number of studies which focus on normative data in nationally representative groups of adolescents. One meta-analysis of objective quantitative sleep parameters, from childhood to old age in healthy individuals, has calculated based on 18 studies including a total of 1360 children total sleep time, assessed by polysomnography (the gold standard physiological measure of sleep) [28]. The authors showed that total sleep time varied widely among the studies: from 8 to 9 hours and 30 minutes at the age of 11; and from 7 to 8 hours and 40 minutes at 15. These studies, however, were examining slightly different age groups using different measurement techniques in different countries, all of which could contribute to differences in outcome. A nationally representative survey, the American Sleep Academy Task Force, America poll in 2006 has found that teenagers aged 12 and 13 yo (7^th^ and 8^th^ grade) were reporting an average of 8 hours total sleep time on school nights and 15 yo were reporting 7.25 hours. They also showed that sleep during non school days was longer and did not vary much from 11 to 15 yo (over 9 hours of sleep). This study may help to better understand what are normative values of sleep in adolescents in the USA [29].

However, to prevent sleep deprivation in adolescents more extensive knowledge is required about their real sleeping habits in different countries.. These normative data are however essential if we want to define “too short sleep” or “sleep deprivation” and their association with co-morbidities. The aim of this study was therefore to obtain normative data on total sleep time, sleep habits and insomnia in adolescents aged 11 to 15 in France, in a large and nationally representative sample of subjects.

## Methods

### Sampling Design

Analyses were based on a nationally representative, cross-sectional sample of students from France, collected within the cross-national health behaviour in school-aged children survey (HBSC) and conducted under the auspices of the World Health Organisation. By 2010, 42 European and Northern American countries and regions were included [30]–[32]. A common HBSC study protocol standardizes instrumentation, sampling methods, and data collection procedures in each country, with data cleaning and data set construction performed centrally.

As the student sample is clustered into schools and classrooms, it is necessary to take into account the effects of clustering on the potential homogeneity of data within selected schools and classrooms. Students aged 11, 13, and 15 years were targeted (mean ages: 11.5, 13.5, and 15.5 years) broadly covering the onset and the middle years of adolescence, when decision-making begins [30].

In order to reaching the international requirement to obtain a 95% confidence interval for a proportion of 50% by age group, and under the hypothesis of an answer rate of 80% a total of 367 schools were randomly sampled at national level, and 347 were included in the French 2010 HBSC sample. This sample was stratified according to three criteria: the urbanity level of the city where the school was located, the class academic level and the private/public status of the school. It was therefore built to represent the socio-demographic characteristics of the French national population of this age group [30]–[32].Data were collected using standardized self-completion questionnaires administered in classes during Spring 2010.

### Ethics

Participation was anonymous and voluntary; consent was obtained from both parents and students. The study protocol was approved by the Ministry of Education ethical national review board for surveys involving people and data management (Comité d'éthique du Minsitère de l'Education Nationale) and the French National Commission on Information Technology and Liberties (Commission Nationale Informatique et Libertés (CNIL)).

A personal letter was sent to each parent (carer) to explain the study and get their approval. If they consent they did not have to answer (passive consent). If they did not consent they had to complete a written document and send it to their child's school with prepaid mail. Ethics committees approved this consent procedure.

Children had also to give their written consent, putting their signature on a name list which was provided by the teachers. This process allowed confidentiality of the questionnaires. If they did not consent, they were allowed to do other school activities in a separate room from children participating to the study to manage silence and self concentration. Ethics committees approved this consent procedure.

### Measurements

Sections investigating sleep habits were introduced in the French HBSC for the first time in 2010. From previous studies [30]–[32] specific sleep investigations were tested and piloted in France along with the standard HBSC questionnaire on health, health behaviour and their determinants and broader context. These sleep-specific measurements were inspired from validated tools recommended for the assessment of sleep in adolescence such as enquiring specifically about and separating sleep on school nights to sleep on weekends and using diaries to keep a log of sleep hours [33]–[34]. As our survey was designed to be made on a single day, we used single item sleep habit reports to assess total sleep time. The following information was collected:

**An estimate of the total sleep time (TST)** during the schooldays (TSTS) and during non schooldays (week-ends or non school days) (TSTN) was based on the following questions:
  1. ‘When you have/don't have class the following day, at what time do you usually go to bed?’ with 13 possible answers for each question: “not after 9 p.m.”, around 9.30 p.m., around 10 p.m., around 10.30 p.m.; around 11 p.m., around 11.30 p.m., around midnight, around 12.30 a.m., around 1 a.m., around 1.30 a.m., around 2 a.m., around 2.30 a.m., “3 a.m. or later”. When the subjects ticked off “not after 9 p.m.”: we entered 9 in our calculation and when they ticked off “3 a.m. or later”: we entered 3.
  2. ‘When you have/don't have class the following day, at what time do you usually wake up?’ with 15 possible answers for each question “not after 5 a.m. around 5.30 a.m., around 6 a.m., around 6.30 a.m., around 7 a.m., around 7.30 a.m., around 8 a.m., around 8.30 a.m., around 9 a.m., around 9.30 a.m., around 10 a.m., around 10.30 a.m., around 11 a.m., around 11.30 a.m., noon or after noon”. When the subjects ticked off “not after 5 p.m.”: we entered 5 in our calculation and when they ticked off “noon of after noon”: we entered 12.
  3. ‘Usually how long does it take for you to fall asleep?’ with 5 possible answers: “less than 10 minutes”, “from 11 to 20 minutes”, “from 21 to 30 minutes”, “from 31 to 40 minutes”, “more than 40 minutes”. When a subject ticked off “less than 10 minutes” we entered 10, “from 11 to 20 minutes” we entered 15, “from 21 to 30 minutes” we entered 25, “from 31 to 40 minutes” we entered 35, “more than 40 minutes” we entered 40.
  TST was defined as the difference between the time at which the participant went to bed and the time of the day they wake up, discounting the time needed to fall asleep.

**Sleep problems were defined as follows:**
  “Sleep initiating problems”: There is a lack of consensual definition of insomnia in children. In early childhood it is important to take account of and use parents as witnesses [35]. However teenagers are more likely to answer to the same definition than adults, which was proposed by international consensus groups [36]–[37]. Based on these we considered that our subject had “sleep initiating problems” when they answered “every night” or “several times per week” to the question “Have you had trouble falling asleep during the last 6 months?” They were considered to have daytime consequences of poor sleep when they answered “more than 4 times a week” or “2 or three times a week” to the question: “Do you feel tired when you wake up on days you have class?”.
  “Sleep debt”: Despite the absence of a consensual definition for sleep debt in adolescents, most authors and the National sleep Foundation in the USA consider that a “2 hours debt” was sufficient enough to evoke sleep debt in teenagers [38]–[41]. We therefore defined sleep debt in our subjects as a difference between TSTN and TSTS for over 2 hours.
  ‘Too short sleep’: In adults, subjects sleeping less than 6 hours during the weekdays are usually considered “short sleepers” and may potentially be at higher risk of developing co morbidities [17]. In teenagers, based on previous recommendations and observations, we considered that sleeping 7 hours or less was “too short” [21]–[28], [38].

### Statistical Analysis

Data collected were weighted using national data concerning sex, age and student academic levels distribution, in order to being representative of the French student teenager population. Analysis Data management and statistical analysis were performed using the R software (version 2.12.1). Reported differences were significant at the 0.01 level or less. The Holm-Bonferroni method has not been used for multiple comparisons. Collected data were analyzed in the total population and separately for boys and girls, as significant associations were initially found between gender and TST. An Analysis of Variance (ANOVA) was performed to test the difference between the mean outcome scores across the different age groups. Comparisons between groups of total sleep time were also made using student ANOVA for continuous data and chi-squared test for categorical data.

## Results

Response rates at school were 93.5%. Out of 9251 students (4643 boys and 4608 girls participated to the study), students' non response was due to parental refusal (7.7%), student refusal (1.2%) and absence of the student the day of the survey (7.3%). Non respondents were not significantly different from respondents for age and sex. They were significantly different regarding socio-demographic profiles. Those figures are in line with those observed in other European countries in such school surveys. Due to the design of the study, this sample was representative of the general group of adolescents of France.

### Total sleep time (Table 1)

**Table 1:** Total Sleep time during schooldays (TSTS) and non schooldays (TSTN), and percentage of teenagers with sleep debt, too short sleep and sleep initiating problems.

Total (n = 9251)	11 years	12 years	13 years	14 years	15 years	Total	ANOVA comparing each age group to the other one
Average TSTS (hours and minutes) (SD)	9h26 (1 min)	9h01 (1 min)	8h39 (2 min)	8h18 (2 min)	7h55 (2 min)	8h41 (1 min)	***
Average TSTN (hours and minutes) (SD)	10h17 (2 min)	10h10 (2 min)	9h56 (2 min)	9h56 (2 min)	9h44 (2 min)	10h01 (1 min)	***
% of children with Sleep debt (TSTN – TSTS>2 hours)	16.0	20.4	26.5	32.6	40.5	27.0	***
% of subjects with TSTS≤7 hours	2.6	5.6	10.5	16.2	24.6	11.7	***
% of subjects with sleep initiating problems	16.5	17.1	20.0	21.8	20.8	19.2	**
***Boys (n = 4643)***	***11 years***	***12 years***	***13 years***	***14 years***	***15 years***	***Total***	
Average TSTS (hours and minutes) (SD)	9h26 (2 min)	9h03 (2 min)	8h44 (2 min)	8h19 (2 min)	7h59 (2 min)	8h45 (1 min)	***
Average TSTN (hours and minutes) (SD)	10h04 (3 min)	9h57 (3 min)	9h48 (3 min)	9h44 (3 min)	9h34 (3 min)	9h50 (1 min)	***
% of children with Sleep debt (TSTN – TSTS>2 hours)	12.5	16.0	22.2	2.0	32.7	22.1	***
% of subjects with TSTS≤7 hours	3.0	6.2	8.9	17.0	23.7	11.4	***
Sleep initiating problems insomnia	14.8	14.9	17.3	17.9	15.9	16.2	P = 0.367
***Girls (n = 4608)***	***11 years***	***12 years***	***13 years***	***14 years***	***15 years***	***Total***	
Average TSTS (hours and minutes) (SD)	9h26 (2 min)	8h59 (2 min)	8h34 (2 min)	8h18 (2 min)	7h52 (2 min)	8h40 (1 min)	***
Average TSTN (hours and minutes) (SD)	10h30 (3 min)	10h23 (3 min)	10h05 (3 min)	10h08 (3 min)	9h54 (3 min)	10h12 (1 min)	***
% of children with Sleep debt (TSTN – TSTS>2 hours)	19.6	24.5	30.9	37.1	47.8	31.9	***
% of subjects with TSTS≤7 hours	2.1	5.1	12.3	15.4	25.4	11.9	***
Sleep initiating problems	18.3	19.3	22.9	25.7	25.5	22.3	***

*** *: p<0.001;*

** *: p<0.01;*

Legend: TSTS = total sleep time during schooldays, TSTN = total sleep time during schooldays, SD = standard deviation, n = number, y = years, p = statistical significance (95%), min = minutes, h = hours.

TSTS decreased continuously and significantly from an average of 9 hours (h) 26 minutes (min) (3 min) among 11 year-olds to an average 7 h 55 min (3 min) among 15 year-olds (p<0.001). Table 1 shows that, in both genders, TSTS decreased with an average of 20 min per year and a total difference mean of 1 h 31 min between ages 11 and 15 (p<0.001).

TSTN also significantly declined in the total group of students, from 10 h 17 min (4 min) at age 11 to 9 h 44 min (4 min) at age 15 (p<0.001). The decline is not as steep as TSTS, but significant.

The average TSTN was greater in girls (10 h 12 min (2 min) vs. 9 h 50 min (2 min) in boys, p<0.001).

### Sleep initiating problems

Sleep initiating problems were reported by a significantly higher percentage of young subjects of the total group when age increased: 16.5% of the 11 yo, vs. 20.8% of the 15 yo (p<0.001 among age groups). In boys no significant difference was however found among ages. In girls the difference was highly significant (18.3% of the 11 yo, vs. 25.5% of the 15 yo; p<0.001 among age groups).

### Sleep debt and too short sleep

Sleep debt, which was defined by a TSTN –TSTS difference greater than two hours increased significantly and steadily between 11 and 15, concerning 16.0% of 11 yo and up to 40.5% of those aged 15 yo (p<0.001). Sleep debt was higher in girls in each age group and globally (31.9% of girls vs. 22.1% of boys, p<0.001).

‘Too short sleep’ (TSTS<7 h) also increased gradually and significantly as it was reported by 24.6% of the 15 yo vs. 2.6% of the 11 yo (p<0.001). This occurred in both genders (23.7% of the 15 yo vs. 3.0% of the 11 yo boys (p<0.001) and respectively 25.4% and 2.1% among girls (p<0.001)).

## Discussion

Despite increasing evidence that sleep debt is a common behaviour in adolescents and several epidemiological studies made across the planet on adolescents sleep debt and recovery [21]–[27], there were, to our knowledge, except one important telephone call survey made in 2006, in the USA [28], very few epidemiological surveys producing normative data on sleep length in a national representative group of teenagers and none in France. Such data would be a helpful tool in helping parents and kids carers on health education and prevention rules for preserving sleep in adolescents. Recommendations currently given to caregivers concerning sleep requirements in adolescence have increased the awareness of public health authorities in the field of education and children's health [38]–[40]. According to the US National Institute of Health, sleep requirements for teenagers and preteens are estimated to be between 10 hours for 12 yo and 8.5 hours for 18 yo [40]. According to the World Health Organisation office in Europe, adolescents need between 9 and 10 hours of sleep per day [41]. One major finding of our study is that teenagers are commonly sleeping less than is expected. We found that children at 12 yo are more likely to sleep 9 hours and those of 15 yo; 8 hours. An even more severe reduction of TST in teenagers was previously reported by report of the American Sleep Academy Task Force, America poll in 2006 [29]. Children were interviewed by telephone (in the presence of their caregivers who were also interviewed) [42]. It was found that teenagers aged 12 and 13 yo (7^th^ and 8^th^ grade) were reporting an average of 8 h of total sleep time on school nights and 15 yo were reporting 7.25 h. They also showed that sleep during non school days was longer and did not vary much from 11 to 15 yo (over 9 h of sleep). Our study confirms, in an epidemiological setting, that total sleep time decreased severely during adolescence. Several authors have previously reported and reviewed this sleep reduction as it seems contradictory considering adolescents' high sleep requirements at this crucial period of their lives [21]–[27], [42]. This sleep reduction was indeed found in different cultures like Korea (with a mean sleep duration on weekdays of 5 h 42 min (1 h 0 min) per day and on weekends of 8 h 24 min (1 h 36 min) per day) [24]. We already reported the same kind of results in Brazil, in Texas (with 20% of kids sleeping less than 6 h during schooldays), in Taipei and in Iceland [21]–[22], [25]–[26]. Sleep loss through adolescence is indeed not driven by a reduction in sleep requirements but arises from a convergence of biologic, psychological, and socio-cultural influences [43].

Another strong point of this study is the collaboration with the HBSC which covered a representative sample of students and delivered confidential questionnaires to the students. Parents' testimonies are well known to not reflect the real sleep habits of teenagers. Closing the bedroom door and saying “goodnight” does not ensure that the child falls asleep immediately [44]–[45]. The older children become, the less parents accurately capture night time habits [45]. It was the strength of our study: to directly and confidentially interview children (without the presence of their parents) on their sleep and sleep problems. Moreover we carefully designed and piloted questions that had not been previously extensively used in this population to capture accurate times for going to sleep and awakening as well as sleep latency, on schooldays and non schooldays evenings. Interviewing students rather than their parents may explain the differences found between our results and those published in other recent studies, which based on parents' reports. In a representative sample of 1916 Canadian preadolescents, authors found, based on weekly assessments over 6 months that the evolution of weekday time in bed (TIB) decreased from a mean of 10 h and 29 min (32 min) per day at 10 yo to 9 h and 29 min (37 min) per day at 13 yo. They therefore considered as short sleepers participants who slept less than 10 hours per day [27]. In the Sleep in America survey on Teens, parents curiously reported an increase in TIB from 11 yo (9 h50 min), to 15 yo (10 h 43 min), which reflects the discrepancy between parents and adolescents perceptions of time in bed and sleep time [42]. As mentioned by several authors the majority of parents are unaware of their adolescents' sleeping patterns [44]–[45].

In this study we also attempted to clarify how sleep debt or too short sleep may be defined in adolescents. Chronic sleep deprivation in adults is known to be associated with poor vigilance, cognition and mood as well as promotes anxiety and a higher risk of accidents [12]–[14]. Chronic ‘too short sleep’ (less than 6 hours per 24 hours) leads to an increased risk of diabetes, raised BMI and cardiovascular diseases [16]–[21]. In adolescents several recent studies showed similar links: ‘too short sleep’ was associated with raised BMI, diabetes, depression, fatigue and suicidal thoughts [23]–[27], [42], [46]–[49]. However these last studies failed to agree on consensual definitions of ‘too short sleep’ and ‘sleep debt’ in adolescents. In our study, we proposed a definition for ‘sleep debt’ in adolescents (defined by a TSTN –TSTS difference greater than two hours) and ‘too short sleep’ (TSTS<7 hours)) based on naturalistic and non clinical databases. We also considered that sleeping less than 7 hours may be considered as “too short sleep”. The data revealed an impressive increased in students concerned by these two problems: sleep debt concerned 16% of the 11 yo group and 40.5% of the 15 yo whereas ‘too short sleep’ was complained of by 2.6% of the 11 yo and 24.6% of the 15 yo. However we understand that the cutoff of 7 hours may be controversial. Thus, additional rationale for the use of this cutoff in this population is warranted. Laboratory studies data regarding sleep extension, grades, assesment of sleepiness by objective tests such as Multiple sleep latency test (MSLT) would be helpful to validate this cutoff.

However we acknowledge that there are several limitations to our study. This study is a non longitudinal, cross sectional survey. Sleep was assessed once during the year and could not be considered as representative of sleep habits over the year. Moreover we used subjective questionnaires to assess sleep and acknowledge that polysomnography is the only objective gold standard method to assess sleep quality and quantity [37]. Several studies carried out in adolescents used actigraphy as well as questionnaires. However several of these studies showed that the sleep logs and questionnaires used to assess sleep patterns in adolescents used in our study are also validated tools to accurately estimate sleep duration in teenagers [22], [43]–[45]. We recognize that we only focussed on total sleep time with no consideration for physical or psychological diseases which could influence individual and collective sleep. This was not the aim of this study as we prioritised collecting normal data on sleep patterns in a representative sample of the adolescents. However future studies should aim to focus on the determinants and correlates which may influence total sleep time in this crucial period of the life.
